# PD-L1 Targeting Immune-Microbubble Complex Enhances Therapeutic Index in Murine Colon Cancer Models

**DOI:** 10.3390/ph14010006

**Published:** 2020-12-23

**Authors:** Daehyun Kim, Seung Soo Lee, Hyungwon Moon, So Yeon Park, Hak Jong Lee

**Affiliations:** 1 Department of Nano Science and Technology, Graduate School of Convergence Science and Technology, Seoul National University, Seoul 08826, Korea; daehyun.kim@nanoimgt.com; 2 Department of Radiology, Seoul National University Bundang Hospital, 82 Gumi-ro 173, Bundang-gu, Seongnam 13620, Korea; 3 IMGT Co., Ltd., Seongnam 13605, Korea; 2slee88@gmail.com (S.S.L.); hyungwon.moon@nanoimgt.com (H.M.); soyeon.park@nanoimgt.com (S.Y.P.); 4 Bio-MAX Institute, Seoul National University, 1 Gwanak-ro, Gwanak-gu, Seoul 08826, Korea

**Keywords:** focused ultrasound, microbubbles, cancer immunotherapy, immune checkpoint inhibitors, immune-related adverse effects

## Abstract

Cancer immunotherapy has revolutionized the way different neoplasms are treated. Among the different variations of cancer immunotherapy, the checkpoint inhibitors targeting the programmed cell death protein 1 (PD-1)/programmed death-ligand 1 (PD-L1) axis have been validated and are currently used in the clinics. Nevertheless, these therapeutic antibodies are associated with significant side effects and are known to induce immune-related toxicities. To address these issues, we have developed an immune-microbubble complex (IMC) which not only reduces the toxicities associated with the antibodies but also enhances the therapeutic efficacy when combined with focused ultrasound. The concept of IMCs could be applied to any type of antibody-based treatment regimens to maximize their therapeutic potential.

## 1. Introduction

The advent of cancer immunotherapy has shifted the cancer-treatment paradigm. Since the goal of immunotherapy is to empower the body’s immune system to kill cancer cells [1], it practically does not involve toxic materials or surgery against body mechanisms, thereby minimizing side effects [2,3]. Among potential cancer immunotherapies, methods such as application of specific antibodies, improving antigen presentation, immune checkpoint blockade therapies, and therapies against the tumor microenvironment (TME) are currently being evaluated in clinical trials. Furthermore, combinations of such therapies to improve efficacy are also being evaluated [3,4,5,6,7]. 

Tumor cells utilize their physiological methods to evade immune response for their survival. On the surface of the tumor cells, programmed death ligand 1 (PD-L1) is normally upregulated, allowing them to interact with the programmed death 1 (PD-1) on the surface of the T cells that induce suppression of immune response upon the PD-L1/PD-1 binding. During this interaction, CD80 [8,9], a member of the immunoglobulin superfamily that provides important antigen-nonspecific costimulatory signals for maximum immune responses, is also involved [10], recruiting the Src homology 2 domain-containing protein tyrosine family phosphatases (SHPs). The recruitment of SHPs cause TCR reverse mechanisms of phosphorylation signals so that T cells become incapable of releasing granzymes and perforins regardless of the recognition of the major histocompatibility complex (MHC) I [11]. These include stimulation of regulatory T cells, promotion of T cell apoptosis, and prevention of the activation of effector T cells [12].

Although the blockade of PD-L1 has shown some clinical promises, there are still issues that need to be addressed with this approach. First, therapeutic antibodies against the PD-L1 are rarely used alone because the therapeutic effects are not as significant [13,14]. As such, checkpoint inhibitors are often used in combination with chemotherapeutic agents to maximize the therapeutic potential [15,16,17], which can elicit potential chemo-related side effects [18]. Furthermore, despite the concept of boosting one’s immune system, the application of PD-L1 inhibitors are not without side effects themselves [19]. An increasing amount of reports on the immune-related adverse effects (irAEs) and hypersensitivity are now becoming available [20]. It has been reported that approximately 10 to 20 percent of patients treated with PD-L1 inhibitors have shown irAEs [21,22]. In addition, instances of fetal hypersensitivity have also been reported upon the administration of PD-L1 monoclonal antibodies into preclinical animal models, inducing irreversible damage and death [23]. As such, there is a strong need for the development of agents and/or methods that can minimize the xenogeneic toxicities while maximizing therapeutic efficacy to be met.

The combination of checkpoint inhibitors with focused ultrasound (FUS) is being actively investigated to complement cancer immunotherapy [23,24]. High-intensity FUS, either by itself or in combination with microbubbles, has been used to ablate local tumors by generating thermal effects at the focal region [23]. In addition, the FUS-mediated mechanical fractionation of tumors physically alters the tumor microenvironment, enhancing the release of chemokines or cytokines from the tumors which leads to priming of the dendritic cells against the released tumor antigens and also increased infiltration of immune cells into the system [25,26,27]. To the added benefit, ultrasound-assisted cavitation of microbubbles can temporarily increase the size of vascular fenestrations, allowing enhanced extravasation of therapeutic agents into the interstitial space for desired effects [28,29].

To capitalize on these features and to circumvent irAEs, we have developed a new type of microbubble (MB) delivery system called the immune-microbubble complex (IMC), in which phospholipid microbubbles are covalently labeled with PD-L1 antibodies. This way, the targeting and therapeutic efficacy of PD-L1 are maintained, while the potential immunogenic responses are alleviated by making it difficult for the immune cells to recognize the antibody through polyethylene glycol “stealth” mechanisms and partial blockage of the Fc region due to the antibody-MB conjugation. Besides, the application of ultrasound will ensure that (1) targeted tumors are partially/fully fragmented by mechanical forces to improve antigen presentation, (2) cavitation by the IMCs themselves will enhance extravasation into the tumor region, and (3) only upon the ultrasound exposure will the MB “burst open”, allowing the antibodies to become free and interact with their receptors. In this paper, we were able to minimize antibody-related fatalities in the mice cohorts and maximized PD-L1 monotherapy using the combination of IMC and therapeutic ultrasound.

## 2. Results

### 2.1. Characterization of MBs and IMC

**Figure 1:**
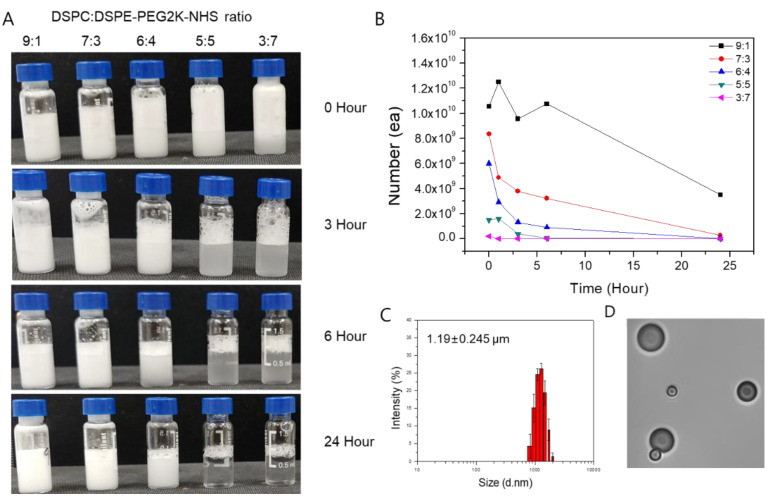
The yield, size, and stability of microbubbles (MBs) with different molar ratios of the phospholipids. (**A**) Synthesized MBs with various molar ratios of DSPC:DSPE-PEG2K-NHS. MBs with a higher DSPC ratio retain their structure for up to 24 h, while a higher ratio of DSPE-PEG2K leads to MB instability. (**B**) MBs were counted under the light microscope. Similar to the visual inspection, MBs with 9:1 DSPC:DSPE-PEG2K ratio had the highest count at the time of synthesis and 24 h post-synthesis. (**C**) The average size of the synthesized MBs with 9:1 DSPC:DSPE-PEG2K ratio was 1.19 ± 0.245 μm. (**D**) A picture of the MBs was taken under the light microscope.

The MBs and IMCs were synthesized based on the phospholipid thin-film hydration method. To maximize the yield and stability of MB, we first examined combinations of different phospholipid molecules at various molar ratios (Figure 1). Based on the experimental data, we found that the 9:1 1,2-distearoyl-sn-glycero-3-phosphocholine(DSPC) to 2-distearoyl-sn-glycero-3-phospho-ethanol-amine-N-[succinyl(polyethylene glycol)-2000] (DSPE-PEG2K-NHS) molar ratio resulted in the maximum MB production. The yield for the 9:1 ratio was 1.055 × 10^10^ MBs, while 8.35 × 10^9^, 6.01 × 10^9^, 1.51 × 10^9^, and 2.1 × 10^8^ MBs were formed for 7:3, 6:4, 5:5, and 3:7 molar ratios of DSPC to DSPE-PEG2K-NHS, respectively. Furthermore, the 24-h stability was highest for the 9:1 ratio as well, with 3.52 × 10^9^ MBs remaining after 24 h compared to 7:3, 6:4, 5:5, and 3:7 ratios that had 2.8 × 10^8^, 3.2 × 10^7^, 2.9 × 10^5^, and 2.1 × 10^5^ MBs remaining, respectively. As such, the 9:1 molar ratio of DSPC to DSPE-PEG2K-NHS was used throughout this study. Using the dynamic light scattering techniques, the average size of the MBs at the 9:1 DSPC:DSPE-PEG2K-NHS ratio was measured to be 1.19 ± 0.245 μm, with the zeta potential of −3 ± 1.21 mV.

**Figure 2:**
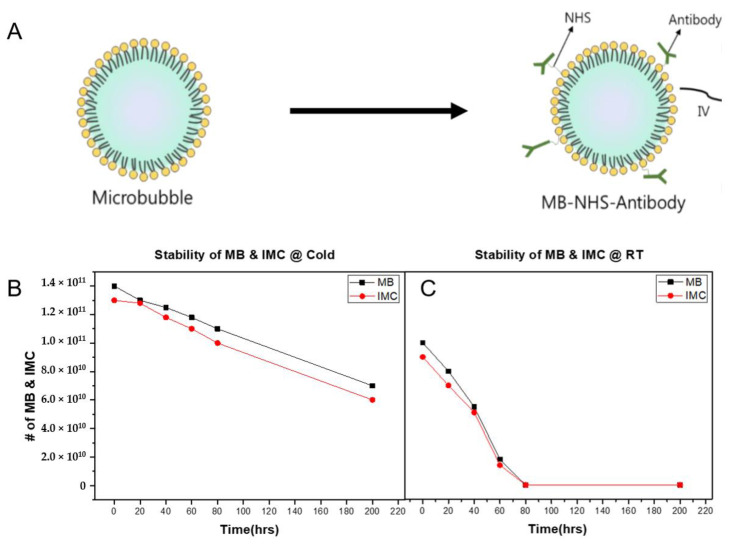
Synthesis of the immune-microbubble complex (IMC) and their stability. (**A**) The schematics of conjugating antibodies onto the surface of MBs using amine-NHS crosslinking. (**B**) The stability of the MBs and IMCs at 4 °C over 200 h. For both MBs and IMCs, up to 50% of the initial amount remained viable. (**C**) The stability of the MBs and IMCs decreased significantly at room temperature conditions, suggesting that higher temperature was associated with their decay.

Next, to produce IMC, the PD-L1 antibodies were conjugated onto MBs by exploiting the NHS functional group on the MB surface. The average size of IMCs was 1.06 ± 0.312 μm with zeta potential values of −2 ± 0.75 mV, suggesting that the synthesized IMCs have comparable physical characteristics to the parent MBs. The conjugation efficiency was also evaluated using the Bradford assay by calculating the number of antibodies that remain in the supernatant post conjugation. Because 100-fold molar excess of NHS was present, the conjugation efficiency of antibodies was near 100%. The stability of IMC after antibody conjugation was then evaluated. At 4 °C, both the MBs and IMCs remained relatively stable, with over 70% of them remaining intact after three days (Figure 2). Up to 50% of the initial amount of MBs and IMCs remained viable after 200 h as well, suggesting the structural stability at lower temperatures. However, the stability of both MBs and IMCs had decreased dramatically at room temperature, with only 50% of MBs and IMCs remaining intact after 40 h, and close to none after 72 h.

### 2.2. Confocal Image of IMC 

**Figure 3:**
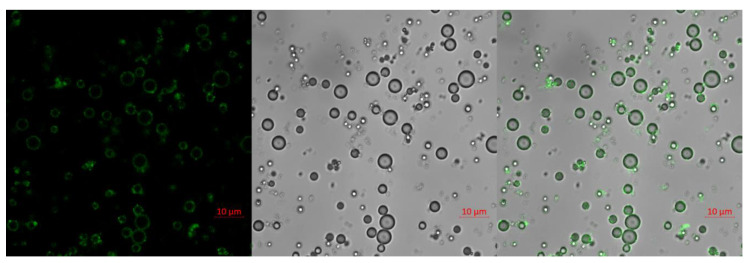
Confocal images of FITC-labeled IMC. Scale bar: 10 μm.

To confirm the antibody conjugation and visualize the IMCs, fluorescein isothiocyanate (FITC)-conjugated antibodies were conjugated onto the surface of MBs, similar to the protocol for conjugation of the anti-PD-L1 antibody. The fluorescence-labeled antibody-MB complex was examined with an LSM710 confocal microscope at ×1000 magnification. A strong FITC fluorescence, corresponding to the borders of the MBs in differential interference contrast images, was observed, confirming that the antibodies could be successfully conjugated onto the surface of the MBs (Figure 3).

### 2.3. Improved Toxicological Profiles of IMC over the PD-L1 Antibodies In Vivo

**Figure 4:**
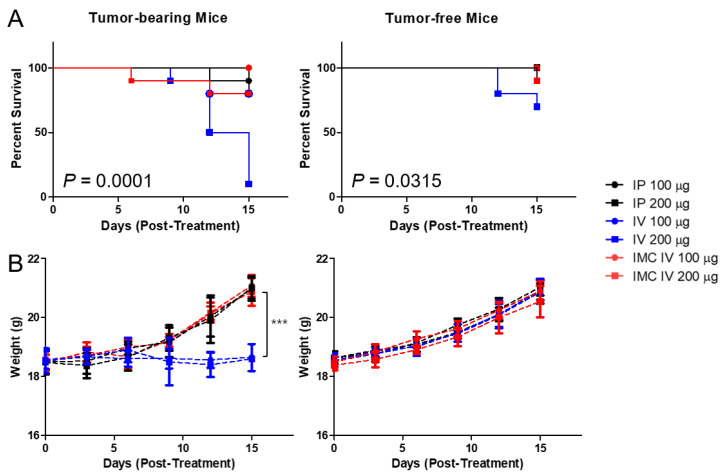
The survival analysis of anti-PD-L1 antibody administered CT26-wt tumor-bearing mice and the evaluation of the effects of the dose and different routes of administration. (**A**) The intravenous injection of PD-L1 antibodies at higher doses (200 μg) in tumor-bearing and tumor-free mice showed increased adverse effects, as 90% and 30% of the mice died within two weeks after injection, respectively. (**B**) The tumor-bearing mice that were injected with the PD-L1 antibodies intravenously at both high and low doses showed reduced weight gain, which may suggest potential adverse effects induced by these antibodies. All the other mice in both the tumor-bearing and tumor-free groups showed a steady increase in the average body weight over the two weeks. IP—intraperitoneal injection; IV—intravenous injection; Values are mean ± SD (*n* = 10 per group). *** *p* ≤ 0.001.

According to the report from Mall et al. [23], repeated administration of PD-L1 monoclonal antibodies induced severe hypersensitivity reactions in orthotopic 4T1 murine mammary carcinoma models. During our experiments, we also discovered that injecting high doses of xenogeneic PD-L1 antibodies in BALB/c mice carrying subcutaneous CT26 carcinoma led to unexpected deaths. We hypothesized that these sudden, unexpected deaths were potentially associated with irAEs, and first tried to establish whether the administration of antibodies were indeed the cause of the mortalities. We prepared two cohorts of mice, one bearing CT26 colon carcinoma and the other not. We have also compared two different routes of administration commonly observed in drug treatment, intraperitoneal (IP) and intravenous (IV). Two doses (100 and 200 ug of antibodies per injection, equivalent to approximately 4 and 8 mg/kg) were administered in bolus every three days, five times. It was observed that the cohort without tumors had a higher overall survival rate than those with tumors (44/60 for tumor-bearing cohort vs 56/60 for the control group at day 15) (Figure 4). Unsurprisingly, the IV route had a lower survival rate than the IP route as 26/40 mice survived the treatment regimen by day 15 in the IV and 37/40 in the IP group. We speculated that because the direct entry into the circulation from the IV route has higher bioavailability than the IP route, a rapid systemic immune response against the xenogeneic antibody can be triggered, causing sudden deaths. Furthermore, similar to Mall et al.’s speculations, the high inflammatory nature of certain tumors induces accumulation of immune cells, thereby promoting a strong immune response against the PD-L1 antibody. 

To overcome the irAEs, we have designed IMC, in which PD-L1 antibodies are conjugated onto microbubbles to enhance circulation and to alleviate problems related to toxicity. Polyethylene glycol chains on the MB surface provides the “stealth” mechanism, further preventing the macrophages from recognizing and mustering an immune response against them. When the IMCs were administered intravenously into the mice cohorts at the same concentrations, the toxicities of the antibodies decreased dramatically, as 18/20 (10/10 for 100 μg and 8/10 for 200 μg injected group) of the tumor-bearing and 19/20 of the control mice (10/10 for 100 μg and 9/10 for 200 μg injected group) survived the repeated dose schedules without noticeable signs or symptoms, compared to the cohorts receiving the unmodified PD-L1 antibodies, with survival rates of 9/20 (8/10 for 100 μg and 1/10 for 200 μg) for the tumor-bearing and 17/20 (10/10 for 100 μg and 7/10 for 200 μg) for the control group (*n* = 10 per group). This pattern was evident in the weight changes of the mice as well. For the groups that received the PD-L1 antibody intraperitoneally or IMC through the IV route, their body weights gradually increased over the 15 days regardless of tumor-bearing or not. Nevertheless, the mice bearing tumors that had received intravenous injections of the PD-L1 not only had higher mortality but also a significant decrease in the weight gains as well. Based on the experimental data, the survival rate was lowest in the tumor-bearing mice that received PD-L1 antibodies intravenously, while those receiving IMCs had significantly improved.

### 2.4. Inhibition of Tumor Growth by the IMC-FUS Combination Therapy

**Figure 5:**
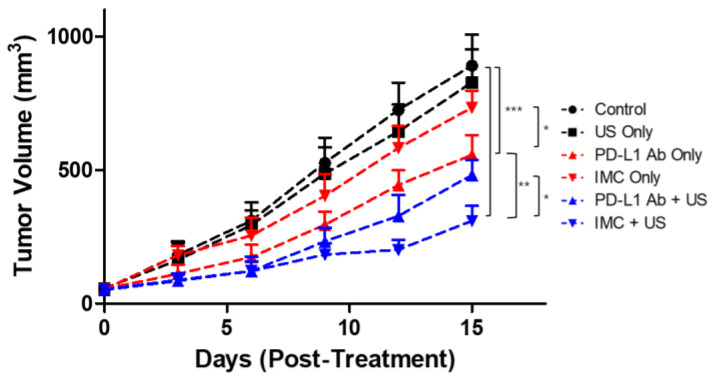
The therapeutic efficacy of PD-L1 targeting protocols against CT26 colon tumors. The control, ultrasound (US) only, and IMC only groups did not show any regression of the tumor after treatment protocols were employed. As expected, the administration of PD-L1 antibody was effective in retarding the tumor growth. The therapeutic effects were maximized with the combination of IMC with focused ultrasound treatment, from which the synergistic effects of both the enhanced localization of the PD-L1 antibody and the cavitation induced by the focused ultrasound can be expected. Values are mean ± SD (*n* = 5). * *p* ≤ 0.05, ** *p* ≤ 0.01, *** *p* ≤ 0.001.

Next, the therapeutic efficacy of the PD-L1 antibody was evaluated. To determine the appropriate dosing schedule and the route of administration, CT26-wt colorectal cancer mouse models were prepared. Different amounts of the PD-L1 antibodies (200 and 100 μg) as well as the route of administration (IP vs IV) were compared. Based on the preliminary data, we found that 200 μg of antibodies injected intravenously had the strongest tumor suppression (data not shown). Furthermore, because five complete treatments over fifteen days were lethal to the tumor-bearing mice receiving intravenous anti-PD-L1 antibody injections, we have hypothesized that the mice would only be able to tolerate up to three treatments without showing significant weight changes based upon the survival analysis. Based on these premises, we prepared another set of CT26 tumor-bearing mice to evaluate the therapeutic efficacy of the combinatorial therapy using FUS and IMC (Figure 5). A total of six experimental groups were prepared: (i) the negative control group injected with saline, (ii) US only, (iii) PD-L1 antibody only, (iv) IMC only, (v) PD-L1 antibody + FUS, and (vi) IMC + FUS. There was no statistical difference between the tumor volumes of the control group, US-treated group, and those that received IMC only (890.1 ± 116.7, 827.5 ± 124.7, and 732.5 ± 64.2 mm^3^, respectively). The PD-L1 antibodies were somewhat effective in retarding the tumor growth (556.5 ± 74.6 mm^3^) when compared to the control group. Maximum therapeutic efficacy was observed for the groups that received a combination of PD-L1 antibody (480.5 ± 58.1 mm^3^) or IMC with FUS treatment (309.7 ± 56.4 mm^3^). The latter was especially efficient in suppressing the tumor growth as the IMC-FUS combination treatments were significantly better than the combination of PD-L1 antibody with FUS.

### 2.5. Immunohistochemical Staining of the Tumor Confirms Enhanced Localization of PD-L1 Antibodies

**Figure 6:**
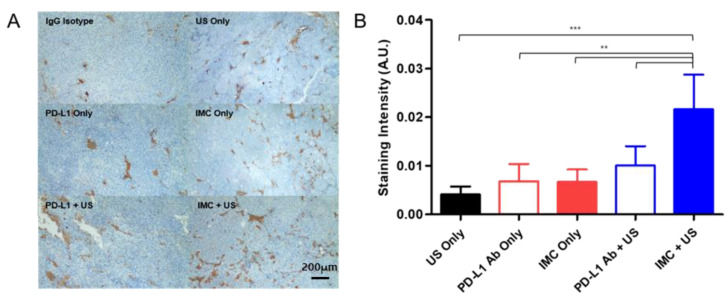
Immunohistochemical evaluation of different treatment groups. (**A**) Representative pictures for each treatment group after staining are presented. (**B**) The intensity of PD-L1 antibody staining from these pictures were analyzed using ImageJ. The staining intensity of the IgG isotype was subtracted from each group to account for the non-specific binding and background signals. Values are mean ± SD (*n* = minimum 3 for each group). ** *p* ≤ 0.01, *** *p* ≤ 0.001.

The localization of the PD-L1 antibodies at the tumor site upon the different treatment protocols were evaluated with immunohistochemistry methods. Similar to the efficacy studies, a total of six experimental groups were prepared: (i) negative control receiving an intravenous injection of the generic IgG antibody, (ii) PD-L1 antibody, (iii) FUS only, (iv) IMC only, (v) PD-L1 antibody + FUS, and (vi) IMC + FUS. 24 h after each cohort received their respective treatment protocols, the mice were euthanized, and the tumors were collected for immunohistochemical staining. An enhanced localization of the PD-L1 antibody was observed in the group that had received IMC and was treated with FUS protocols (Figure 6), while the other groups showed relatively lower levels of the PD-L1 antibody bound onto the tumor surface. 

## 3. Discussion

As third-generation cancer therapeutic agents, immune checkpoint inhibitors that are involved in T-cell regulation such as PD-L1 antibodies have been successfully validated in preclinical models and are currently used in clinical settings against different types of cancers. To reduce the immunogenicity, therapeutic antibodies in clinical applications are humanized to remove the potential immunological responses [30]. Nevertheless, despite the efforts to minimize the toxicity, humanized monoclonal antibodies may still induce potential and serious adverse immune-related complications [31,32]. We observed similar immunogenic responses as we were developing a syngeneic mouse model bearing CT26 colon cancer cells to evaluate immune checkpoint inhibitor therapies using rat-derived anti-mouse PD-L1 antibodies.

Dose-limiting toxicities remain as one of the biggest challenges associated with drug delivery regardless of the type of molecule being used [33,34]. As Mall et al. reported, repeated intraperitoneal injections of the PD-L1 antibodies to the mice bearing orthotopic 4T1 murine mammary carcinoma induced fatal hypersensitivity reactions [23]. Our experimental data also showed that higher doses of antibody injection induced fatality regardless of the route of administration (Figure 4). When the repeated dose was increased to 400 μg per injection, the fraction of mice surviving decreased even more (survival of 8/10 for those injected with 200 μg antibody → 7/10 for those injected with 400 μg antibody in the tumor-bearing mice cohort, *p* = 0.6004 and 10/10 for mice for those injected with 200 μg antibody → 8/10 for those injected with 400 μg antibody in the tumor-free mice cohort, *p* = 0.146; data not shown). The intravenous injections of the PD-L1 antibodies were even more lethal, as 9/10 of the tumor-bearing mice receiving 200 μg antibody per injection had died within two weeks after injection, with significant reductions in weight gains.

To alleviate the associated adverse effects, the presentation of active pharmaceutical ingredients in carriers such as liposomes has become a standard practice to enhance the pharmacokinetic/dynamic profiles in vivo. Doxil (liposomal doxorubicin) and Abraxane (albumin-bound paclitaxel) are two examples of such nanoformulations used in clinics that have greatly increased the therapeutic index of the parent drugs by extending their circulatory half-life and reducing the associated immunotoxicities [35,36,37]. As such, we hypothesized that by presenting the therapeutic PD-L1 antibodies in a nanoformulation, we could expect similar improvements in the therapeutic index and avoid the immune adverse effects. IMCs have not only met these criteria to minimize adverse effects, but they also introduce an additional dimension to improve the therapeutic efficacy—the concept of cavitation-mediated drug delivery.

**Figure 7:**
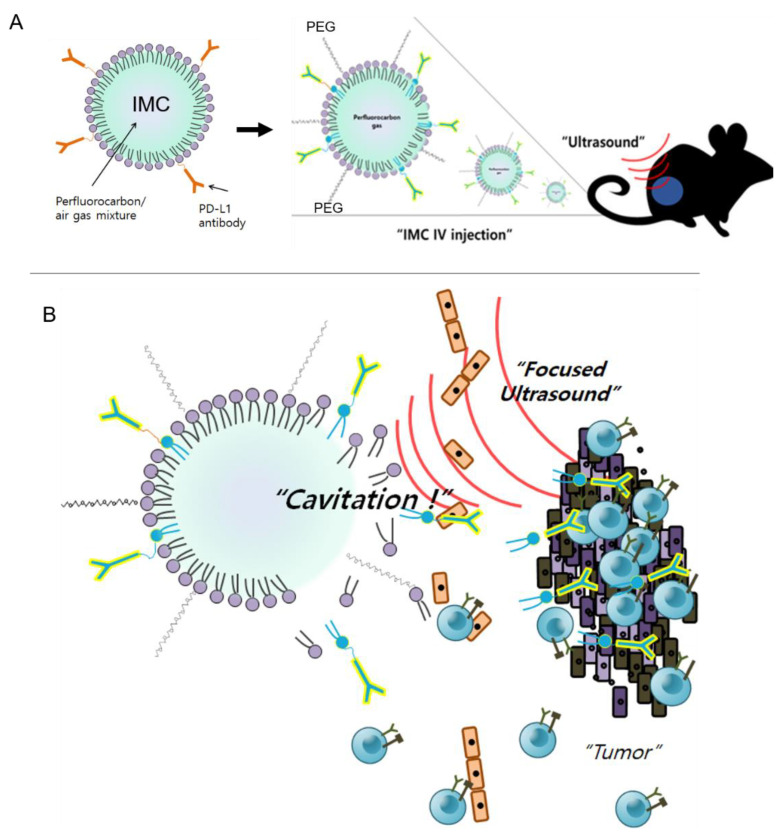
The proposed mechanism of action for IMC and the combination therapy. (**A**) IMCs are injected intravenously into the tumor-bearing mice. Because they are PEGylated and larger in size, they are less likely to induce immune responses compared to the individual antibodies. (**B**) IMCs are stimulated with ultrasound, which causes acoustic cavitation as well as their breakdown, allowing the PD-L1 antibodies to better access the tumor. The blocking of the surface PD-L1 on the tumors allows cytotoxic T cells to recognize the tumor cells and destroy them.

During the last decade, the use of ultrasound with MBs to enhance local drug delivery has been well-studied in the preclinical and clinical settings [38,39] against brain diseases [40], breast cancer [41], and pancreatic cancer [42]. At the focal point, the converged ultrasound beams cause cavitation of the injected MBs, which temporarily disrupt the endothelial linings and increase drug extravasation into the interstitial space for enhanced therapeutic effects [39]. Likewise, when IMCs were combined with the ultrasound treatment against CT26 tumors, the anti-cancer effects were maximized (Figure 5). On the other hand, IMCs by themselves did not have strong anti-cancer effects as the tumor growth was comparable to that of the PBS-injected (control) group, suggesting that an additional trigger is essential for the IMCs to become effective. Based on experimental evidence, we propose the following mechanism of action for IMCs and the IMC + FUS combination therapy (Figure 7): (1) IMCs, unlike the parent PD-L1 antibody, is PEGylated and much larger in size, which enhances their half-life and prevents potential immune responses. (2) IMCs are stable at room temperature for at least 24 h (Figure 2), so their structure would remain relatively intact in circulation and prevent the conjugated PD-L1 antibody from binding onto the PD-L1 expressed on the surface of CT26 tumors. (3) Upon the focused ultrasound treatment, IMCs undergo cavitation to increase extravasation, and eventually break down to expose the individual antibodies which then bind to the surface of PD-L1-expressing tumors. Subsequently, CD8 + T cells can recognize and remove the tumor cells. 

The role of FUS in the tumor microenvironment remains to be elucidated for this study. FUS treatment, by itself or in combination with MBs, causes mechanical fractionation of the tumor tissues at the focal point and triggers the release of tumor antigens to the microenvironment [43]. Enhanced tumor antigen release potentiates dendritic cell maturation, which in turn triggers priming of the T cells and immunological responses against the tumor cells [44,45]. While we speculate that similar mechanisms are responsible for the results obtained in this study, we were not able to validate the results with immunological evaluations. In the near future, we plan to follow up on this study by confirming the changes in cytokine expressions, T cell infiltrations, as well as potential tumor rechallenged experiments.

## 4. Materials and Methods

### 4.1. Preparation of the Lipid Microbubbles

Microbubbles (MB) were synthesized based on the phospholipid thin-film hydration method. 1,2-distearoyl-sn-glycero-3-phosphocholine(DSPC) and 1,2-distearoyl-sn-glycero-3-phospho-ethanolamine-*N*-[succinyl(polyethylene glycol)-2000] (DSPE-PEG2k-NHS, both purchased from Avanti Polar Lipids, Alabaster, AL, USA) were dissolved in chloroform at 9:1 6:4, 3:7, 7:3, and 5:5 molar ratios. Subsequently, chloroform was evaporated with a rotary evaporator (to form a thin phospholipid film). This phospholipid film at a concentration of 0.5 mg/mL was hydrated using 0.01M PBS over the phase transition temperature of DSPC and was dispersed using a bath sonicator. Once completely dissolved, the headspace of empty vials was filled with sulfur hexafluoride gas (SF_6_) for 45 seconds, capped, and was agitated by VialmixTM (Definity, North Billerica, MA, USA) for 45 seconds to generate MBs. MBs were manually counted under a light microscope to approximate their number.

### 4.2. Preparation and Characterization of the IMC

To conjugate microbubbles with antibodies, 1.5 × 10^9^ microbubbles were mixed with 20, 40, 80, 100, and 200 µg of the anti-PD-L1 antibody (BioXcell, Lebanon, NH, USA) for 30 min at room temperature. The unreacted anti-PD-L1 antibody was separated from the IMC by gradient centrifugation at 3000 rpm for 10 min. To confirm the antibody conjugation on the microbubbles, 1 mg of FITC-tagged IgG antibodies were incubated with as-prepared IMC for 60 min at 4 °C. The secondary antibody-conjugated IMC solution was purified by centrifugation for 5 min at 3000 rpm. The supernatant was collected, and the amount of unreacted antibody was measured using the Bradford assay (Thermo Fisher Scientific, Waltham, MA, USA) under the manufacturer’s guidelines. The size distribution and zeta potential of the microbubbles and IMC were measured using a Malvern Zetasizer Nano (Malvern Instrument Ltd., Worcestershire, UK). 

### 4.3. Stability Test of MB and IMC

Microbubbles with different molar ratios of DSPC and DSPE-PEG2K-NHS (9:1, 6:4, 3:7, 7:3, and 5:5, respectively) were used to optimize their stability. After their synthesis, the microbubbles and IMC with different phospholipid ratios were diluted 100-fold with PBS and were counted manually under a light microscope. Measurements were made at 0, 3, 6, and 24 h post microbubble synthesis.

Once the optimal phospholipid ratio for microbubble synthesis was obtained, the long-term stability of prepared microbubbles and the IMC were evaluated by monitoring them for up to 7 days at room temperature and 4 °C. The number of microbubbles and IMC were counted every 24 h manually under the light microscope.

### 4.4. Confocal Imaging

For confocal microscope imaging, the IMC was conjugated with FITC-tagged antibody by adding 50 µg of the antibody to 1 mg/mL of IMC solution and incubating the mixture on a shaker for an hour at room temperature. Subsequently, the IMC-FITC antibody conjugate was centrifuged at 3000 rpm for 5 min to remove unbound antibodies and the samples were placed on a glass slide for microscopy. The fluorescent images of the IMC were obtained using LSM710 (Zeiss, Germany) at a magnification of 1000×, using excitation and emission filters at 490/520 nm, respectively.

### 4.5. Animal Studies

All in vivo protocols were verified according to the guidelines of the Seoul National University Bundang Hospital (Approval Number BA1811-260/079-01). 6- to 8-week-old immunocompetent and immunodeficient female BALB/c nude mice were purchased from Orient Bio (Seoul, Korea) for toxicity and efficacy studies, respectively. The mice were acclimatized for a week before the start of the respective experiments and were maintained at standard conditions in specific pathogen-free (SPF) environments: 25 ± 2 °C temperature, 50 ± 10% relative humidity, and 12h light/12h dark cycle. All mice were fed with sterilized standard mouse chow and water ad libitum.

The experimental groups for the acute toxicity study were designated as follows: (i) PD-L1 antibody (100 µg/intraperitoneal (IP) injection), (ii) PD-L1 antibody (200 ug/IP), (iii) PD-L1 antibody (100 ug/intravenous (IV) injection), (iv) PD-L1 antibody (200 ug/IV), (v) IMC (100 ug/IV), and (vi) IMC (200 ug/IV). Each group received a single bolus injection of 200 μL of the treatment protocols every three days, five times. The conditions and weights of mice were monitored for 15 days following the injection. 

For the efficacy studies, 1 × 10^6^ of CT26-wt cells suspended in Matrigel (Corning, MA, USA) were injected into the right flank region of the nude mice. Tumor sizes were monitored bi-weekly with a digital caliper and the volumes were calculated using the modified ellipsoid formula: width^2^ × length × 0.5. Once the tumor volume reached to 50~70 mm^3^, the mice were randomly sorted for treatment. The experimental groups were defined as follows: (i) Isotype control, (ii) anti-PD-L1 antibody (200 ug antibody concentration), (iii) IMC (200 ug), (iv) FUS only, (v) anti-PD-L1 antibody (200 ug) + FUS, and (vi) IMC (200 ug) + FUS (*n* = 5 per group). Each group received treatments every three days, three times. A pre-clinical FUS system (VIFU 2000^®^, Alpinion Medical Systems, Seoul, Korea) was used for all ultrasound treatments, with the treatment protocols adapted from our previous work [41]. For this study, the FUS conditions were the following: 1.1 MHz frequency, 100 Watts, 100 Hz pulse repetition frequency, 5% duty cycle, 5 s ultrasound exposure per spot, and 2 mm spot distance. The mice were monitored for a week following their respective treatments, and their weights were also recorded at days 0, 3, 6, 9, 12, and 15. 

### 4.6. Preparation of Immunohistochemistry

24 h after respective treatments, tumors were excised and first fixed in 5 L of formalin for 44 min. Subsequently, they were placed in an ethyl alcohol solution for 30 min before being transferred onto another ethyl alcohol solution at a higher concentration. This transfer was repeated 6 times. Following ethyl alcohol fixation, the samples were then placed in xylene solution at a low concentration for 45 min before being transferred onto another xylene solution at a higher concentration for a total of three times. Finally, the samples were then embedded in paraffin wax. The sample processing was performed using Leica Peloris (Buffalo Grove, IL, USA). The embedded tumor samples were cut into 3 μm slices with Leica RM2235 (Buffalo Grove, IL, USA) and incubated in hydrogen peroxide blocks for 10 min. The tumor-bound PD-L1 antibodies were then detected using the UltraVision LP Large Volume Detection System (Thermo Fisher, San Jose, CA, USA) according to the manufacturer’s guidelines. 

### 4.7. Statistical Analysis

Data are expressed as mean ± standard deviation (SD). The survival data were plotted by the Kaplan–Meier method and statistics were calculated using the Mantel-Cox log-rank test. One-way analysis of variance (ANOVA) with Tukey’s post hoc analysis was used to compare experimental groups. ImageJ was used to obtain the intensities of the immunohistochemical stains. GraphPad Prism 5.0 (San Diego, CA, USA) was used for the curation of the graphs and statistical analysis. Probability (*p*) values of < 0.05 were considered as statistically significant.

## 5. Conclusions

In this work, PD-L1 antibody-conjugated microbubbles—termed IMCs—were used in combination with focused ultrasound to treat CT26-wt tumor-bearing colon cancer mouse models. Not only were the IMCs able to alleviate adverse immune responses and fatalities associated with systemic administration of xenogeneic antibodies, but they also enhanced therapeutic efficacy when combined with ultrasound treatment. While the exact immunomodulatory mechanisms remain to be validated, the development of IMCs can serve as a unique way to improve the therapeutic index for antibodies used in clinics today.
